# Supplementary material for: PAX3-FOXO1 uses its activation domain to recruit CBP/P300 and shape RNA Pol2 cluster distribution
Source: Nat Commun. 2023 Dec 15;14:8361. doi: 10.1038/s41467-023-43780-4 (PMC10724205; doi:10.1038/s41467-023-43780-4)
Supplement: Supplementary file 1 — Supplementary Information [file 41467_2023_43780_MOESM1_ESM.pdf]

PAX3-FOXO1 uses its activation domain to recruit CBP/P300 and shape RNA Pol2 cluster distribution

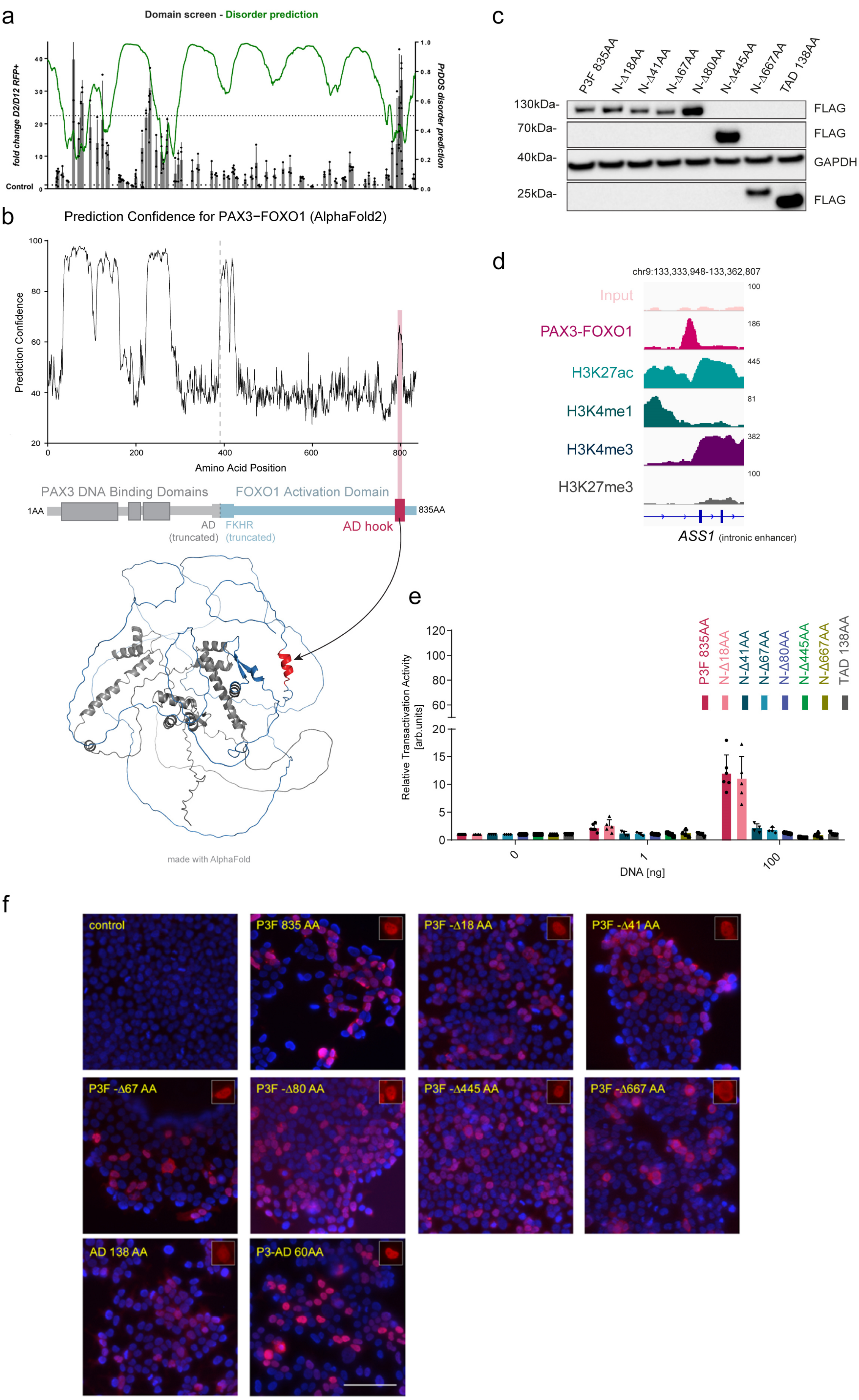

Supplementary Figure S1. The activation domain of PAX3-FOXO1 has a helical structure and its deletion has no influence on subcellular distribution of PAX3-FOXO1.

a PAX3-FOXO1 protein disorder prediction calculated using PrDOS (green line) (www.predictprotein.org). Results of the domain screen from Figure 1B are overlaid as black bars.

b PyMol rendering of the predicted structure of PAX3-FOXO1, generated using AlphaFold2 (https://github.com/deepmind/alphafold). Red highlights a predicted ordered region of FOXO1 that has an alpha-helical structure. The vertical dashed line indicates the breakpoint of the fusion.

c Western blot depicting expression of indicated P3F variants in HEK293T cells. GAPDH was used as a loading control. N - abbreviation for P3F. The Western blot was performed once.

d Genomic track of *ASS1* depicting the binding site of P3F located in intron 9 of *ASS1*.

e Luciferase reporter assays measured 48h after transfection of HEK 293T cells with indicated P3F constructs and using a negative control reporter. The P3F binding site from *ASS1* was mutated for this purpose. Depicted are mean ± SD for each construct (n=6 (P3F 835AA), n=5 (N-Δ18), n=4 (other constructs) independent experiments). Values are normalized to an internal transfection control (Renilla luciferase).

f Immunofluorescence detection of full length and deletion mutants of P3F. 293T cells were transfected with plasmid encoding for indicated Flag-tagged P3F derivate and the protein was detected two days later with an anti-Flag antibody. Insets show one representative cell from each staining. Scale bar, 100 μm.

Source data are provided as a Source Data file.

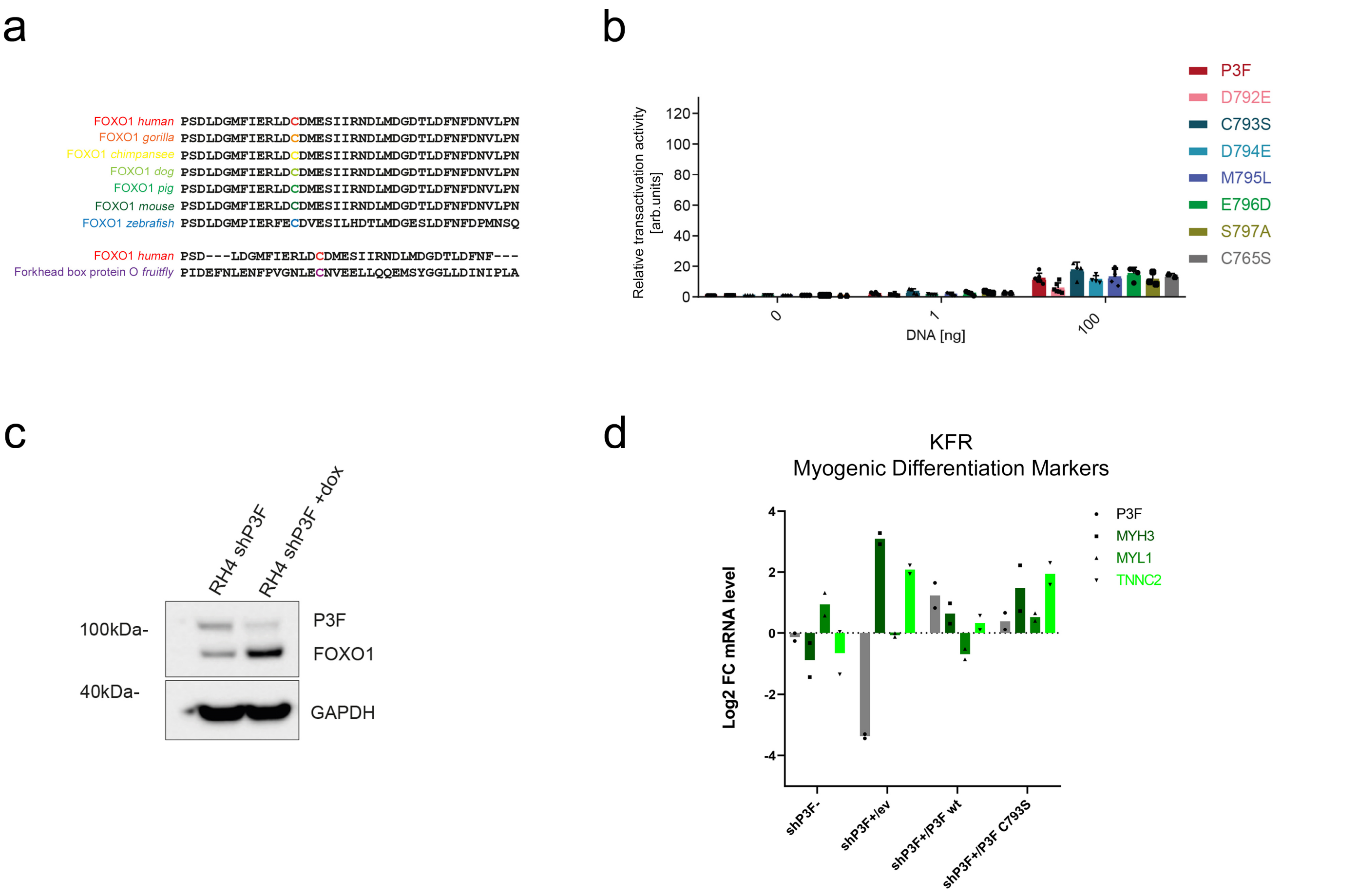

Supplementary Figure S2. Mutation of C793 induces differentiation of FP-RMS cells.
a Alignment of FOXO1 protein sequences from different species around Cys612 (human FOXO1).
b Luciferase reporter assays measured 48h after transfection of HEK 293T cells with indicated P3F constructs and using a negative control reporter. The P3F binding site from *ASS1* was mutated for this purpose. Depicted are mean ± SD for each construct (n=6 (P3F, D792E), n=4 (C793S, D794E,M795L, E796D), n=3 (S797A) and n=2 (C765S) independent experiments). Values are normalized to an internal transfection control (Renilla luciferase).
c Western blot depicting knockdown of endogenous P3F in RH4 cells containing an inducible shRNA. Cells were treated for 48h with 50ng/ml doxycycline. One representative blot from n=2 is shown.
d mRNA levels of different myogenic differentiation markers in KFR cells 48h after silencing of endogenous P3F with a doxycycline-inducible shRNA (shP3F) and transduced with either empty vector (ev), wild type P3F (P3F wt) or P3F carrying a C793S mutation (C793S). mRNA levels of indicated genes were quantified by qPCR and relative mRNA levels were calculated using the ΔΔCT method with GAPDH as reference. Data is normalized to untreated control. (n=2 independent experiments).
Source data are provided as a Source Data file.

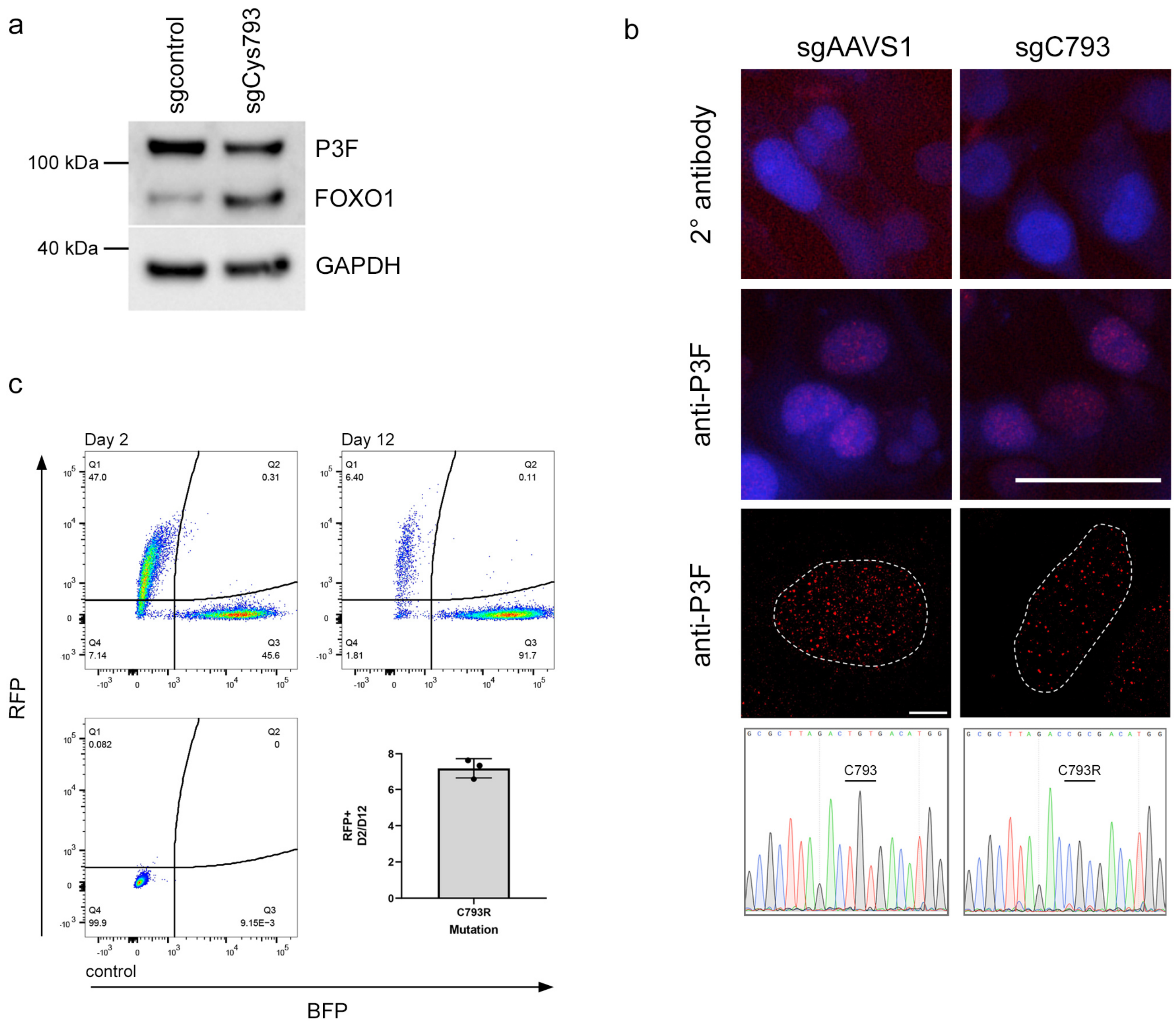

Supplementary Figure S3. Site directed mutagenesis of Cys793 in endogenous P3F affects cell survival but not its nuclear localization.

a Western blot detection of P3F in RH4 cells containing the ABE8e base editor 5 days after transduction with indicated sgRNAs. One representative blot from n=2 is shown.

b Immunofluorescence detection of wildtype and C793 mutant P3F in RH4 cells using a P3F-specific antibody. RH4 cells containing the ABE8e base editor were transduced with either an sgRNA directed against AAVS1 (left panels) or an sgRNA inducing the mutation of C793 by base editing (right panels). Upper two panels show conventional widefield microscopy pictures, third panels show super-resolution microscopy pictures. Dotted lines indicate nuclei. Lower panels show Sanger sequencing chromatograms depicting the sequence region around C793 in endogenous P3F from the same experiment. Scale bar widefield microscopy, 50 µm, Scale bar super-resolution microscopy, 5 µm.

c Competition assay with RH4 cells after mutation of C793 by base editing using an sgRNA in a plasmid with RFP in comparison to control cells transduced with an sgRNA against AAVS1 in a plasmid with BFP. Cell populations were mixed at day 2 after transduction and relative number of RFP- and BFP-positive cells was measured immediately afterwards and at day 12. Scatter plots show data from one representative experiment. Bar graph shows depletion rate of RFP-positive cells at day 12 versus day 2. Depicted is mean ± SD. (n=3 independent experiments).

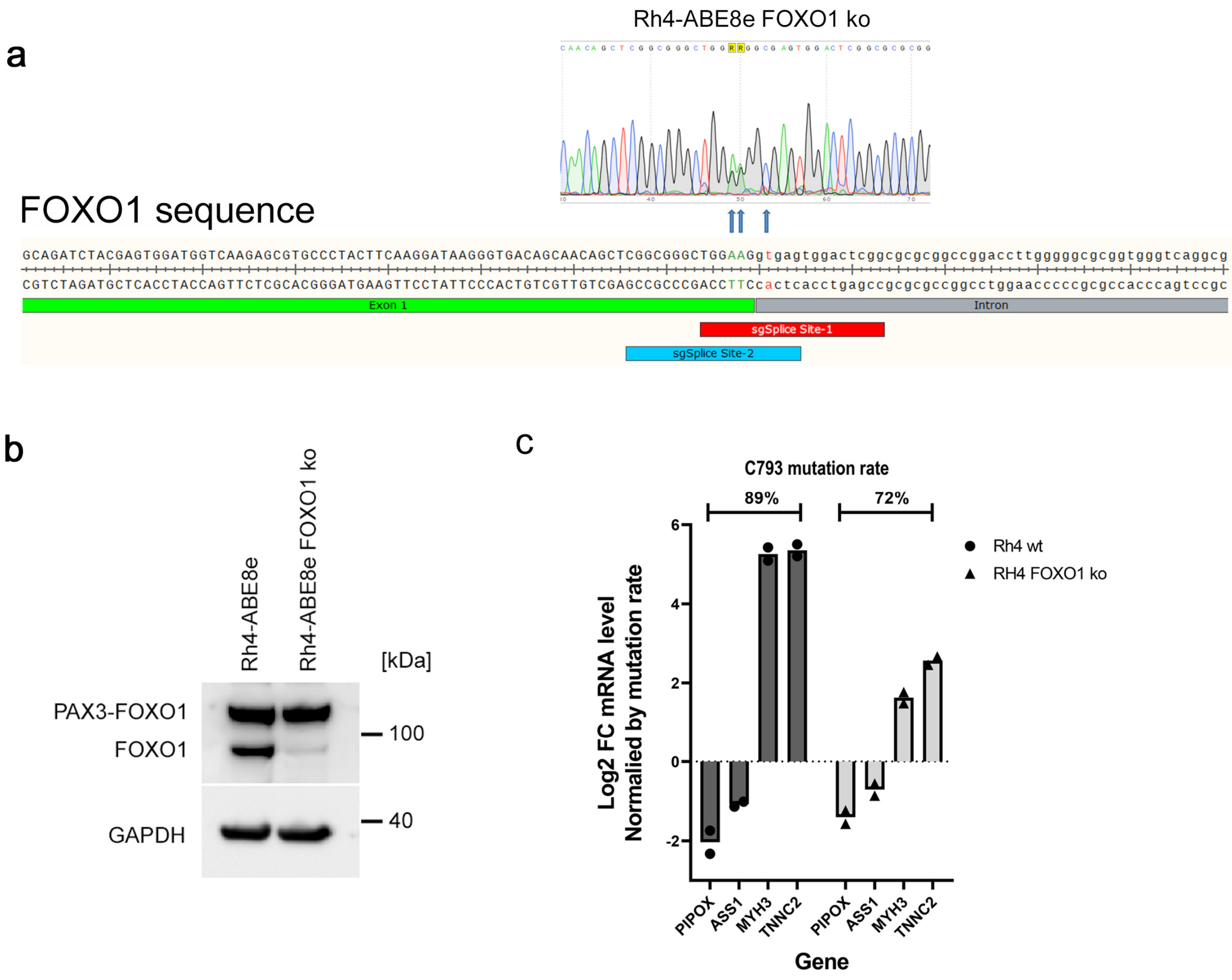

Supplementary Figure S4. Wildtype FOXO1 affects changes in gene expression after inhibition of PAX3-FOXO1.
a Sequence region around the exon/intron boundary of exon 1 in *FOXO1*. Location of sgRNAs used for mutation of nucleotides left (sgSplice site-1) and right (sgSplice site-2) to the splice site are indicated below the sequence. Mutation of nucleotides in RH4 cells containing the ABE8e base editor after transduction with both sgRNAs is shown in the Sanger sequencing chromatogram above the sequence.
b Western Blot detection of PAX3-FOXO1 and FOXO1 in RH4 cells before and after mutation of the splice site of exon 1 of *FOXO1*. One representative blot from n=2 is shown.
c Change of mRNA level of indicated genes after mutation of C793 in parental RH4 cells (left) and in FOXO1-ko cells (right) compared to control cells transduced with an sgRNA against AAVS1. Cells were transduced with sgRNA and RNA was isolated three days later. mRNA levels were detected by TaqMan qRT-PCR. Mutation of C793 was determined by Sanger sequencing in parallel. Fold change in mRNA levels were normalized with mutation levels (n=2 independent experiments).
Source data are provided as a Source Data file.

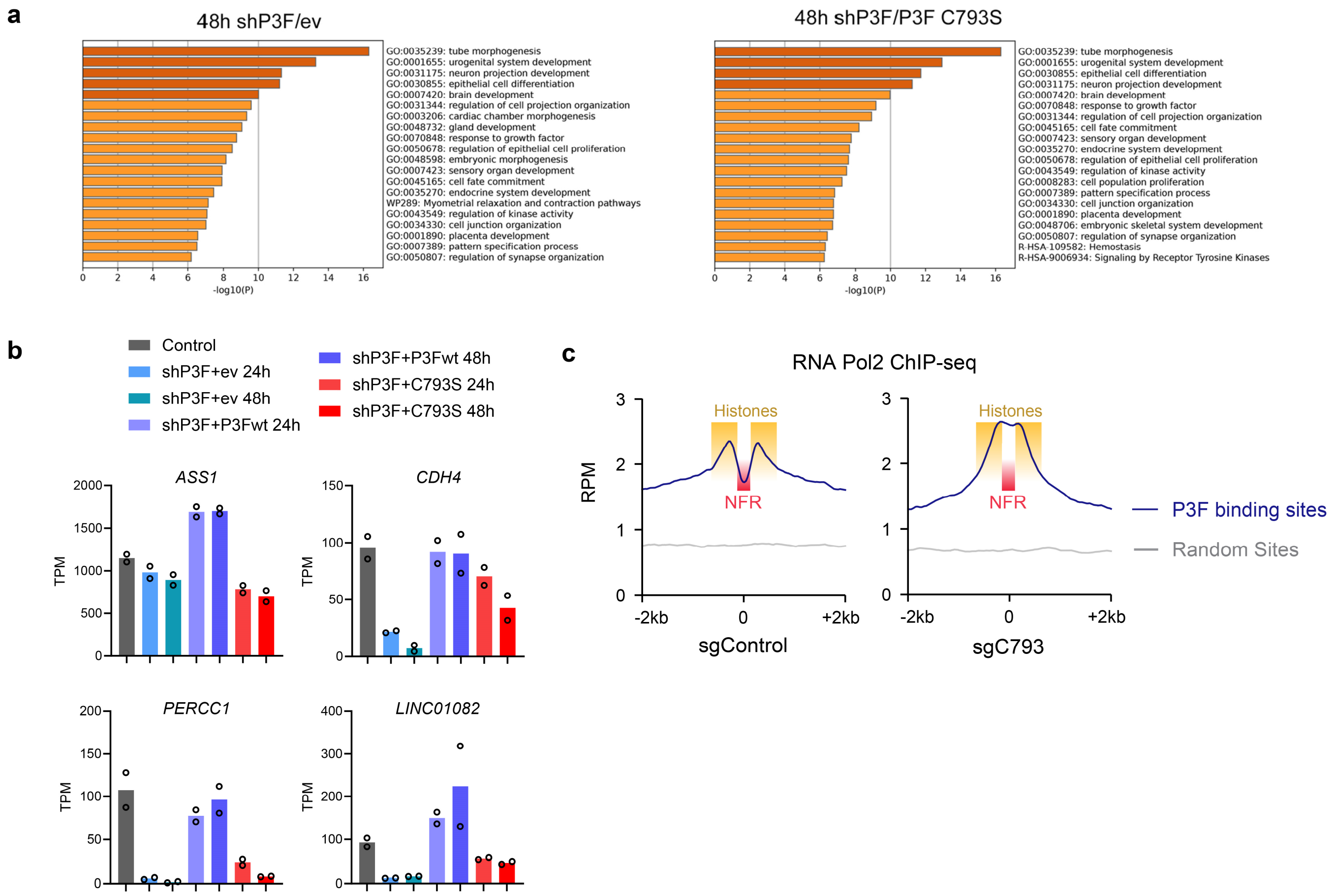

Supplementary Figure S5. C793S mutant PAX3-FOXO1 fails to rescue target gene expression after silencing of endogenous PAX3-FOXO1.

a Top twenty gene ontology terms found enriched for the top downregulated genes after silencing of P3F and rescue with either empty vector (left panel) or C793S mutant P3F (right panel). Metascape was used for the analysis.

b mRNA TPM values of ASS1, CDH4, PERCC1, and LINC01082 across all construct treatments. (data from n=2 independent experiments).

c Profile plots of RNA Pol2 ChIP-seq in adenine base edited RH4 cells expressing either the wild-type or mutant (C793R) P3F at P3F wild-type binding sites compared with random regions in the genome. NFR, Nucleosome free region, highlighted in red and histone co-occupant region highlighted in orange.

Source data are provided as a Source Data file.

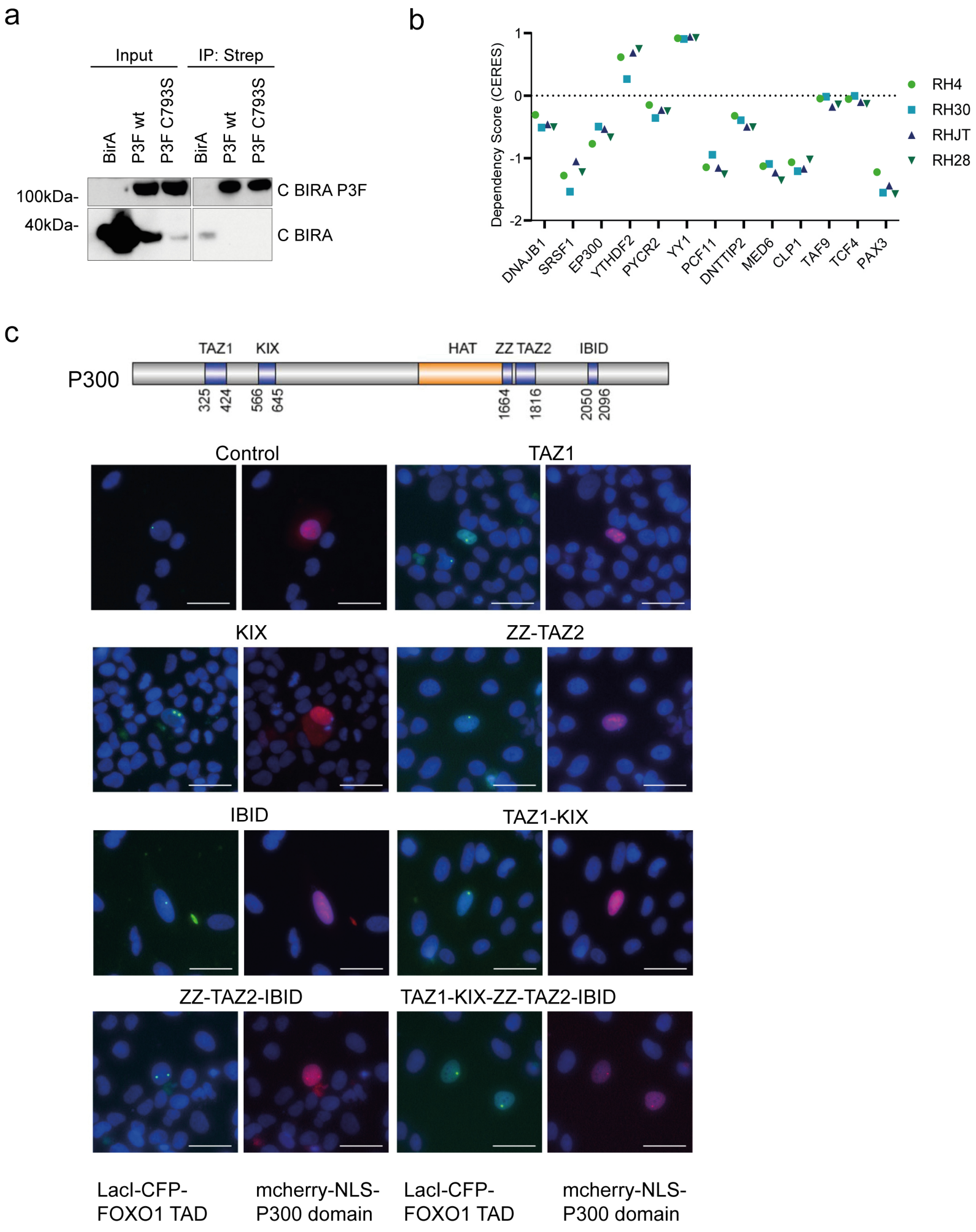

Supplementary Figure S6. Interaction of PAX3-FOXO1 and p300 involves several p300 domains.

a Western blot detection of indicated proteins after streptavidin pull-down from 293T cells. Cells were transfected with BirA or P3F-BirA fusion proteins and biotinylation was induced by incubation with biotin. The Western blot was performed once.

b Tumor dependency scores, calculated by CERES, of the indicated proteins identified by BioID MS in six fusion positive cell lines (CRISPR Avana Public 19Q2, depmap.org). PAX3 was included as reference.

c Recruitment assay performed with the LacI-CFP-FOXO1 CTD fusion protein and different individual or combined p300 domains linked to mCherry. The p300 domain structure is shown in the upper panel. p300 domains tested include TAZ1, KIX, ZZ-TAZ2 and IBID as individual domains and TAZ1-KIX, ZZ-TAZ2-IBID and TAZ1-KIX-ZZ-TAZ2-IBID as fusions. Representative recruitment assay pictures are shown for each tested domain, with CFP signal (left) and mCherry signal (right). Scale bar, 50 µm.

Source data are provided as a Source Data file.

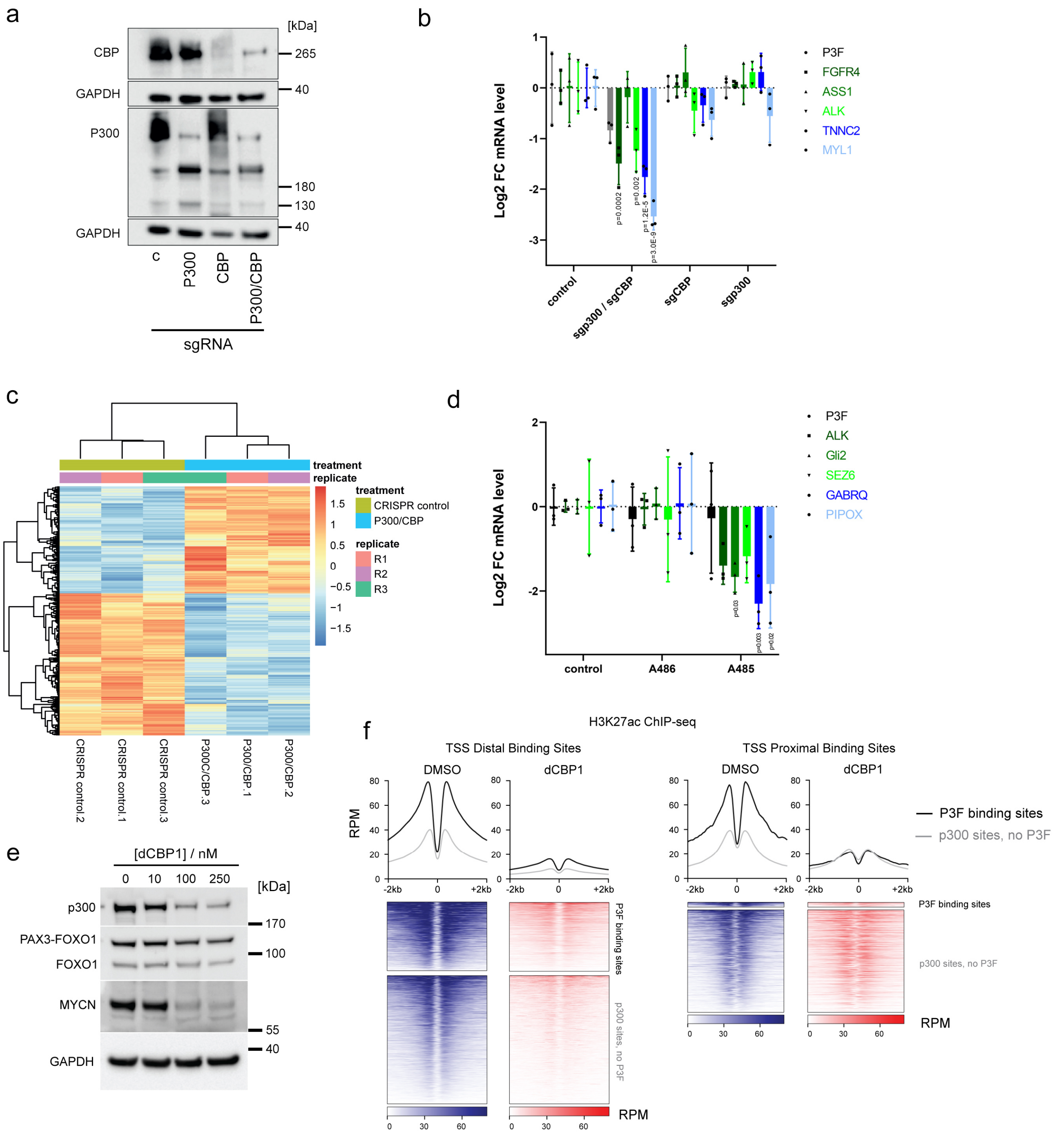

Supplementary Figure 7. Both p300 and CBP are relevant co-factors of PAX3-FOXO1 in FP-RMS cells.
a Western blot depicting individual and combined knockouts of p300 and CBP in RH4-Cas9 cells. One representative blot from n=2 is shown.
b RT-qPCR of P3F target genes and differentiation markers after knockout of CBP and/or p300 in RH4-Cas9 cells (n=3 independent experiments; two-way Anova, Tukey`s multiple comparisons test, comparison to control).
c Unsupervised hierarchical clustering analysis using all genes up- or downregulated after combined knockout of p300 and CBP in RH4-Cas9 cells compared to controls (n=5749; fold change ≥ 1.5 and fdr <5%).
d mRNA levels of indicated genes quantified by qPCR in RH4 cells 6 days after treatment with 1µM of indicated compounds. Genes tested are P3F target genes that depend on p300/CBP expression. Relative mRNA levels were calculated using the ΔΔCT method with GAPDH as reference. Data is normalized to untreated control (n=3 independent experiments; two-way ANOVA, Tukey`s multiple comparisons test, comparison to control).
e Time dependence of protein level changes in key regulators, histone acetylation and TFs PAX3-FOXO1 and MYCN, as shown by dose response Western blot in RH4 cells at 6 hours of dCBP1 treatment. One representative blot from n=2 is shown.
f Heatmap of H3K27ac ChIP-seq performed in RH4 cells treated with DMSO and dCBP-1, split across sites with p300 and P3F binding and sites with just p300 binding and sites which are proximal (+/- 5kb) or distal to gene TSS.
Source data are provided as a Source Data file.

a

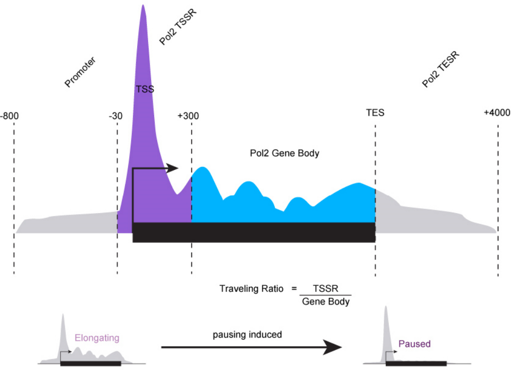

c

gene	DMSO Traveling Ratio	A485 Traveling Ratio	dCBP1 Traveling Ratio
ATOH8	1.46	1.77	2.35
MYCN	3.47	3.73	4.63
MYOD1	3.78	4.20	2.81
SOX8	1.56	1.76	1.87

b

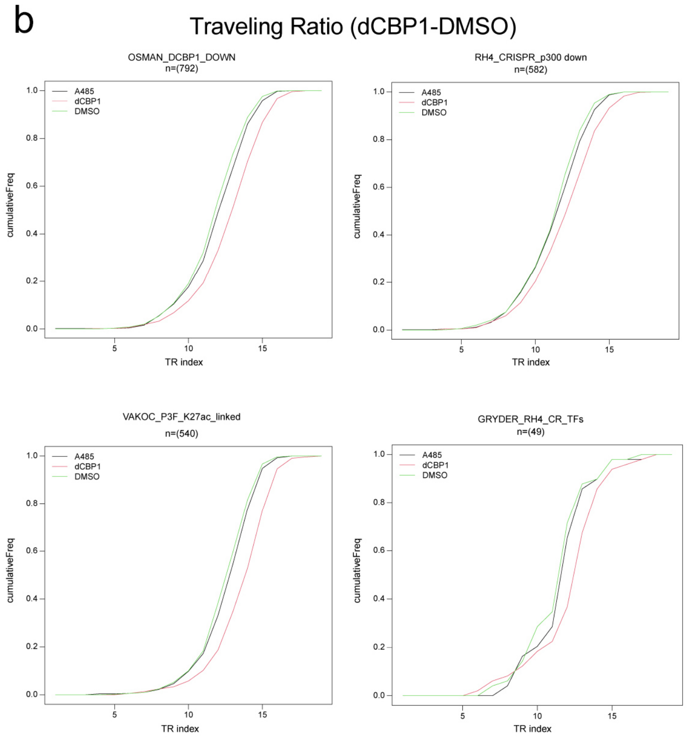

d

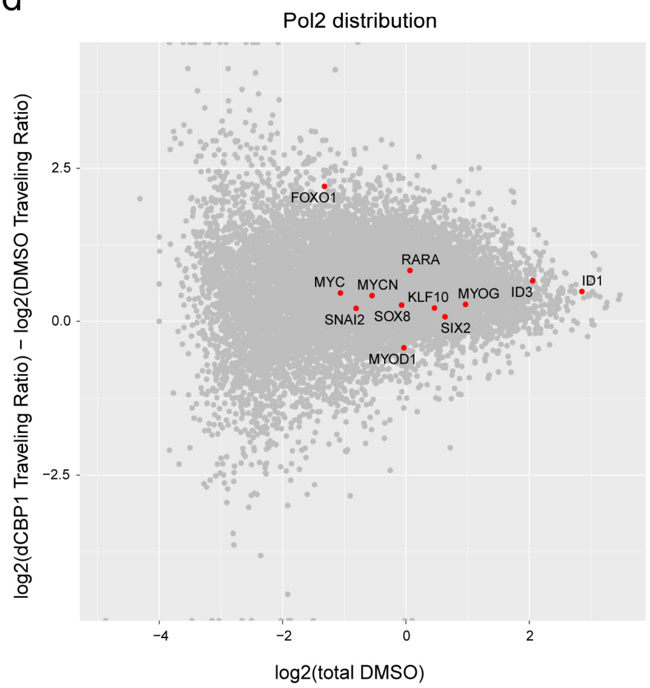

Supplementary Figure S8. Traveling Ratio of Pol2 in response to chemical CBP/p300 disruption.

a Definition of Traveling Ratio (TR) using a schematic Pol2 binding to a gene

b TR trends of gene sets in different drug treatments.

c Pol2 densities of key CR TFs, showing inconsistency in Pol2 pause-release changes upon p300 disruption.

d Scatter plot of DMSO and delta log2FC of TRs after dCBP1 with highlighted CR TF target genes.

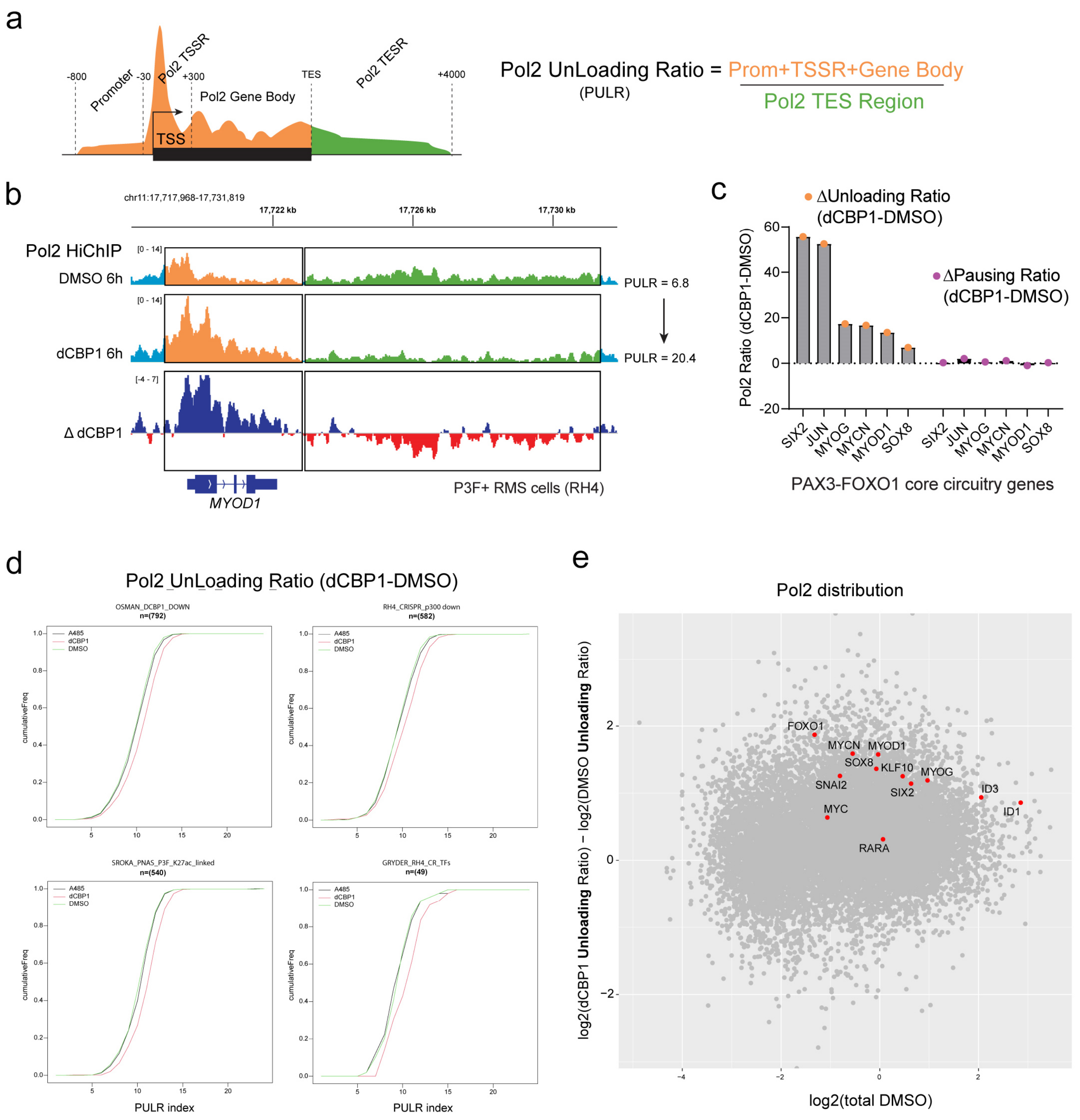

Supplementary Figure S9. Pol2 UnLoading Ratio in response to chemical CBP/p300 disruption.

a Definition of Pol2 UnLoading Ratio (PULR) using a schematic Pol2 binding to a gene.

b Pol2 distribution at *MYOD1*.

c Change in PULR and pausing ratio across PAX3-FOXO1 core circuitry genes following 6h dCBP1 treatment.

d PULR trends of gene sets in different drug treatments.

e Scatter plot of DMSO and delta log2FC of PULRs after dCBP1 with highlighted target genes.

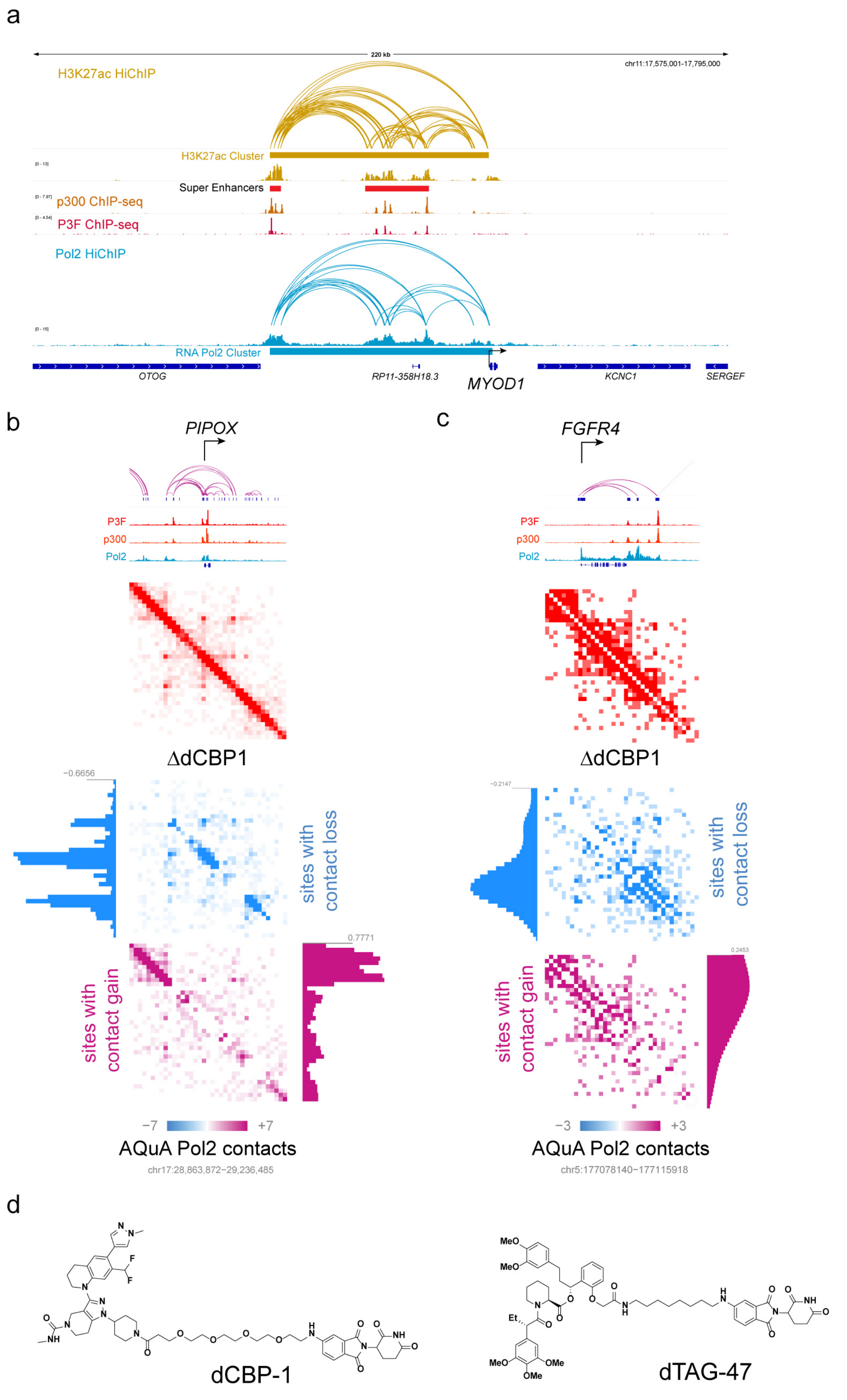

Supplementary Figure S10. RNA Pol2, H3K27ac, p300, and P3F at enhancers of P3F target genes.
a HiChIP and ChIP-seq data demonstrating the overlap of H3K27ac and RNA Pol2 signals at *MYOD1* super enhancers.
b and **c** 3D contact maps of *PIPOX* (b) and *FGFR4* (c) showing AQuA-CPM RNA Pol2 contacts. After 6h dCBP1 treatment, there is an observed loss of long-range contacts with a gain of short-range contacts at a central location.
d Chemical structures for the p300/CBP degrader DCBP1 and the FKBP12 F36V degrader dTAG-47.
