# Supplementary material for: PAX3-FOXO1 uses its activation domain to recruit CBP/P300 and shape RNA Pol2 cluster distribution
Source: Nat Commun. 2023 Dec 15;14:8361. doi: 10.1038/s41467-023-43780-4 (PMC10724205; doi:10.1038/s41467-023-43780-4)
Supplement: Supplementary file 7 — Reporting Summary [file 41467_2023_43780_MOESM7_ESM.pdf]

nature portfolio

Corresponding author(s): Marco Wachtel, Berkley Gryder, Beat Schafer

Last updated by author(s): Nov 2, 2023

Reporting Summary

Nature Portfolio wishes to improve the reproducibility of the work that we publish. This form provides structure for consistency and transparency in reporting. For further information on Nature Portfolio policies, see our Editorial Policies and the Editorial Policy Checklist.

Statistics

For all statistical analyses, confirm that the following items are present in the figure legend, table legend, main text, or Methods section.

n/a	Confirmed	
☐	☒	The exact sample size (*n*) for each experimental group/condition, given as a discrete number and unit of measurement
☐	☒	A statement on whether measurements were taken from distinct samples or whether the same sample was measured repeatedly
☐	☒	The statistical test(s) used AND whether they are one- or two-sided<br>*Only common tests should be described solely by name; describe more complex techniques in the Methods section.*
☐	☒	A description of all covariates tested
☐	☒	A description of any assumptions or corrections, such as tests of normality and adjustment for multiple comparisons
☐	☒	A full description of the statistical parameters including central tendency (e.g. means) or other basic estimates (e.g. regression coefficient) AND variation (e.g. standard deviation) or associated estimates of uncertainty (e.g. confidence intervals)
☐	☒	For null hypothesis testing, the test statistic (e.g. *F*, *t*, *r*) with confidence intervals, effect sizes, degrees of freedom and *P* value noted<br>*Give P values as exact values whenever suitable.*
☒	☐	For Bayesian analysis, information on the choice of priors and Markov chain Monte Carlo settings
☒	☐	For hierarchical and complex designs, identification of the appropriate level for tests and full reporting of outcomes
☒	☐	Estimates of effect sizes (e.g. Cohen's *d*, Pearson's *r*), indicating how they were calculated

Our web collection on statistics for biologists contains articles on many of the points above.

Software and code

Policy information about availability of computer code

Data collection

RNA-sequencing and ChiP-sequencing data collection was performed with Illumina's NextSeq 550 system.
HiChIP sequencing data collection was performed with Illumina's NovaSeq 6000.
Flow cytometry data collection was performed on an BD LSRFortessa instrument with the BD FACSDiva 8.0.1 software.
High content microscopy data collection was performed with an Operetta high content imager and the Harmony 4.9 software.
Super resolution microscopy data collection was performed with a Zeiss Elyra 7 instrument and the Zeiss Zen black 3.5.SR software.
Cell confluence measurements were made with the Incucyte Zoom software
Luminescence data collection was performed with a BioTek Cytation3 Imaging Reader and the BioTek Gen5 2.07.17 software.
Widefield immunofluorescence was performed with Nikon Eclipse Ti2 and the NIS-Elements AR 5.21.02 software.
Mass spectrometry data was collected with a ThermoScientific Orbitrap Fusion Tribrid mass spectrometer.
qRT-PCR data was collected with on an ABI 7900UT fast real-time PCR system with the SDS 2.4 software.
Phase contrast pictures were taken with Nikon Eclipse Ts2 and the DS-L4 controler.
Western blots pictures were taken using a BioRad ChemiDoc Imaging System with the BioRads Image Lab Touch Software 3.0.1.14.

Data analysis

Statistical analysis was performed with Graphpad Prism (v9.3.1) (https://www.graphpad.com/scientific-software/prism/)
Gene tracks were visualized with IGV tools (v2.10.3) (https://software.broadinstitute.org/software/igv/igvtools)
Gene set enrichment analysis was performed with GSEA software version 4.1.0 (https://software.broadinstitute.org/gsea/)
Prediction of protein structure was performed using AlphaFold 2.
Prediction of order/disorder status of proteins was performed using PrDOS (www.predictprotein.org)
Code used throughout paper is available at https://github.com/GryderLab and at https://github.com/GryderLab/rms_additional_code.
Flow cytometry data was analyzed with the software FlowJo V10.8.1.
High content microscopy data was analyszed with Harmony 4.9

Super resolution microscopy data was analyzed with the Zeiss Zen black 3.5.SR software.
Widefield immunofluorescence pictures were processed with the NIS-Elements AR 5.21.02 software.
Cell confluence data was analyzed with the Incucyte Zoom software.
Mass spectrometry data was analyzed with the Scaffold 4.0 software.
qRT-PCR data was analyzed with the RQ manager 1.2.1 software.
Western blots pictures were processed with the Image Lab 6.1.0 software.

For manuscripts utilizing custom algorithms or software that are central to the research but not yet described in published literature, software must be made available to editors and reviewers. We strongly encourage code deposition in a community repository (e.g. GitHub). See the Nature Portfolio guidelines for submitting code & software for further information.

Data

Policy information about availability of data

All manuscripts must include a data availability statement. This statement should provide the following information, where applicable:

- Accession codes, unique identifiers, or web links for publicly available datasets
- A description of any restrictions on data availability
- For clinical datasets or third party data, please ensure that the statement adheres to our policy

The RNA-seq data generated in this study has been deposited in the European Nucleotide Archive (ENA) database with the accession number PRJEB47795. AQuA-HiChIP and ChIP-seq data is deposited with GEO NCBI under the accession number GSE208146. Source data are provided with this paper.

Research involving human participants, their data, or biological material

Policy information about studies with human participants or human data. See also policy information about sex, gender (identity/presentation), and sexual orientation and race, ethnicity and racism.

Reporting on sex and gender	NA
Reporting on race, ethnicity, or other socially relevant groupings	NA
Population characteristics	NA
Recruitment	NA
Ethics oversight	NA

Note that full information on the approval of the study protocol must also be provided in the manuscript.

Field-specific reporting

Please select the one below that is the best fit for your research. If you are not sure, read the appropriate sections before making your selection.

☒ Life sciences ☐ Behavioural & social sciences ☐ Ecological, evolutionary & environmental sciences

For a reference copy of the document with all sections, see nature.com/documents/nr-reporting-summary-flat.pdf

Life sciences study design

All studies must disclose on these points even when the disclosure is negative.

Sample size	No sample size was pre-determined. We have used minimal sample size sufficient to detect biological differences and to perform statistical analysis, if necessary.
Data exclusions	Regions of the genome on the ENCODE blacklist (comprised of, for instance, highly repetitive regions) were excluded from called peaks prior to all downstream analysis (ie, prior to cluster identification in our HiChIP analysis of RNA Pol2 data).<br>For RNA-seq, data was generated from three replicates of each condition, but only two replicates each were included in the final analysis. The reason for the exclusion of the third replicate was an obvious mix up of two samples in this sample set (one was sequenced twice, while the other one was not sequenced.)<br>Otherwise, no data was excluded.
Replication	For ChIP-seq, experiments were performed across similar FP-RMS cell-lines (RH4 and RH5) and compared to prior work done in PMID: 31784732.<br>All other experiments were performed at least twice, in most cases more than this. With exception of the above mentioned RNAseq dataset, all replicates are included.
Randomization	No randomization was performed, since in the experimental setup controls were run side-by-side with the experimental samples.

Blinding

Since in the experimental setup controls were run side-by-side with the experimental samples, blinding was not relevant to our study and no blinding was performed.

Reporting for specific materials, systems and methods

We require information from authors about some types of materials, experimental systems and methods used in many studies. Here, indicate whether each material, system or method listed is relevant to your study. If you are not sure if a list item applies to your research, read the appropriate section before selecting a response.

Materials & experimental systems

n/a	Involved in the study
☐	☒ Antibodies
☐	☒ Eukaryotic cell lines
☒	☐ Palaeontology and archaeology
☒	☐ Animals and other organisms
☒	☐ Clinical data
☒	☐ Dual use research of concern
☒	☐ Plants

Methods

n/a	Involved in the study
☐	☒ ChIP-seq
☐	☒ Flow cytometry
☒	☐ MRI-based neuroimaging

Antibodies

Antibodies used

The following antibodies were used:

For Immunofluorescence:
anti-PAX3-FOXO1 (Clone PFM.2, CancerTools.org, #160866) Used as 1/500 dilution.
anti-p300 (Santa Cruz Biotechnology H-272, sc-8981) Used as 1/250 dilution
anti-Flag M2 (Sigma F9291) Used as 1/500 dilution
anti-rabbit IgG Alexa 568 (ThermoFisher A-11032) Used as 1/500 dilution.
anti-mouse IgG Alexa 594 (ThermoFisher A-11011) Used as 1/500 dilution

For Western Blot:
anti-FOXO1 (Santa Cruz Biotechnology H-128, sc-11350) Used as 1/1000 dilution.
anti-p300 (CellSignaling #54062) Used as 1/1000 dilution.
anti-CBP (CellSignaling #7425) Used as 1/1000 dilution.
anti-HDAC2 (CellSignaling #5113) Used as 1/1000 dilution.
anti-GAPDH (CellSignaling #2118S) Used as 1/1000 dilution.
anti-MYCN (Abcam ab16898) Used as 1/1000 dilution.
anti H3K27Ac (Active Motif #39685) Used as 1/1000 dilution.
anti-Flag M2 (Sigma F9291) Used as 1/1000 dilution.
anti-mouse IgG-HRP (Cell Signaling Technologies #7076). Used as 1/2000 dilution.
anti-rabbit IgG-HRP (Cell Signaling Technologies #7074). Used as 1/2000 dilution.

For ChIP and HiChIP:
anti-RNA Pol2 (Santa Cruz, sc-47701 X). 9 microg antibody per 0.9 microg chromatin was used.
anti-H3K27Ac (Millipore, MABE647 RM172). 2.7 microg antibody per 2.7 microg chromatin was used.

Validation

Validation data for the application immunofluorescence:

anti-PAX3-FOXO1 (Clone PFM.2, CancerTools.org #160866): The PAX3-FOXO1 antibody was validated by groups from the NCI and from the University of Zurich. The data is published in Azorsa et al. , Modern Pathology 2021.

anti-p300 (Santa Cruz Biotechnology H-272, sc-8981): The antibody has been discontinued by the supplier, but there are publications showing its suitabillity for immunofluorescence (e.g. https://europepmc.org/backend/ptpmcrender.fcgi?accid=PMC4093859&blobtype=pdf).

anti-Flag M2 (Sigma F9291) : Validation by Sigma: Detects Flag-tagged proteins in numerous applications including immunofluorescence. Is widely used to detect Flag-tagged proteins. (https://www.sigmaaldrich.com/CH/de/products/protein-biology/protein-sample-prep/flag-purification#detection)

Validation data for the application Western blot:

anti-FOXO1 (Santa Cruz Biotechnology H-128, sc-11350): This antibody was used in numerous publications for Western Blot listed on the SantaCruz website (https://www.scbt.com/p/fkhr-antibody-h-128).

anti-p300 (CellSignaling #54062): Validation by Cell Signaling Technologies: Was shown to detect endogenous levels of human p300 protein. (https://www.cellsignal.com/products/primary-antibodies/p300-d2x6n-rabbit-mab/54062)

anti-CBP (CellSignaling #7425): Validation by Cell Signaling Technologies: Was shown to detect endogenous levels of human CBP protein. (https://www.cellsignal.com/products/primary-antibodies/cbp-d9b6-rabbit-mab/7425)

anti-HDAC2 (CellSignaling #5113): Validation by Cell Signaling Technologies: Was shown to detect endogenous levels of human HDAC2 protein. (https://www.cellsignal.com/products/primary-antibodies/hdac2-3f3-mouse-mab/5113)

anti-GAPDH (CellSignaling #2118S): Validation by Cell Signaling Technologies: Was shown to detect endogenous levels of human GAPDH protein. (https://www.cellsignal.com/products/primary-antibodies/gapdh-14c10-rabbit-mab/2118)

anti-MYCN (Abcam ab16898): Validation by Abcam: Was shown to detect endogenous levels of human MYCN protein (https://www.abcam.com/products/primary-antibodies/n-mycmycn-antibody-ncm-ii-100-ab16898.html)

anti H3K27Ac (Active Motif #39685): Validation by Active Motif: Was shown to detect endogenous levels of human MYCN protein (https://www.activemotif.com/catalog/details/39685)

anti-Flag M2 (Sigma F9291): Validation by Sigma: Detects Flag-tagged proteins in numerous applications including Western Blot. Is widely used to detect Flag-tagged proteins. (https://www.sigmaaldrich.com/CH/de/products/protein-biology/protein-sample-prep/flag-purification#detection)

Validation data for the application ChIP:
anti-RNA Pol2 (Santa Cruz, sc-47701 X): Was used in different publications for ChIP (e.g. https://www.science.org/doi/10.1126/sciadv.aaw5294)

anti-H3K27Ac (Millipore, MABE647, clone RM172): Clone RM172 has been validated for ChIP (https://www.chromatrap.com/uploads/2016-06-30-52-1-900021-chip-validated-h3k27a.pdf)

For HiChIP:
anti-RNA Pol2 (Santa Cruz, sc-47701 X): Was used in different publications for ChIP (e.g. https://www.science.org/doi/10.1126/sciadv.aaw5294).

Eukaryotic cell lines

Policy information about cell lines and Sex and Gender in Research

Cell line source(s)

The alveolar rhabdomyosarcoma cell lines RH4 and Rh5 were both provided by Peter Houghton, Greehey Children's Cancer Research Institute, San Antonio, Texas, USA.
The alveolar rhabdomyosarcoma cell line RMS was provided by Janet Shipley, Sarcoma Molecular Pathology, The Institute of Cancer Research, London, UK.
The alveolar rhabdomyosarcoma cell line KFR was provided by Jindrich Cinatl, Frankfurter Stiftung für krebskranke Kinder, Frankfurt, Germany.
The alveolar rhabdomyosarcoma cell line RH4-PAX3-FOXO1-FKBP12F36V was provided by Dr. Kristy R. Stengel, Albert Einstein College of Medicine.
The alveolar rhabdomyosarcoma PDX-derived models IC-pPDX-104 (origin female RMS patient) and IC-pPDX-35 (origin male RMS patient) were generated in house from a PDX tumor provided by Didier Delattre, Institute Curie in Paris, France.
The alveolar rhabdomyosarcoma model RMS-ZH003 was generated in the lab of Beat Schäfer/Marco Wachtel in Zurich, Switzerland, from a PDX tumor established in the same lab from a female RMS patient.
The myoblast cell line KM155C25Dist was provided by Vincent Mouly, Institut de Myologie, Paris, France.
HEK293T cells and C2C12 cells were both purchased from ATCC.

Authentication

All cell lines were authenticated by short tandem repeat analysis (STR profiling) and positively matched with reference data, if available.

Mycoplasma contamination

Cell lines were regularly tested for Mycoplasma and were Mycoplasma free.

Commonly misidentified lines
(See ICLAC register)

None as far as we know.

Plants

Seed stocks

Report on the source of all seed stocks or other plant material used. If applicable, state the seed stock centre and catalogue number. If plant specimens were collected from the field, describe the collection location, date and sampling procedures.

Novel plant genotypes

Describe the methods by which all novel plant genotypes were produced. This includes those generated by transgenic approaches, gene editing, chemical/radiation-based mutagenesis and hybridization. For transgenic lines, describe the transformation method, the number of independent lines analyzed and the generation upon which experiments were performed. For gene-edited lines, describe the editor used, the endogenous sequence targeted for editing, the targeting guide RNA sequence (if applicable) and how the editor was applied.

Authentication

Describe any authentication procedures for each seed stock used or novel genotype generated. Describe any experiments used to assess the effect of a mutation and, where applicable, how potential secondary effects (e.g. second site T-DNA insertions, mosiacism, off-target gene editing) were examined.

ChIP-seq

Data deposition

☒ Confirm that both raw and final processed data have been deposited in a public database such as GEO.

☒ Confirm that you have deposited or provided access to graph files (e.g. BED files) for the called peaks.

Data access links<br>*May remain private before publication.*	To review the RNA-seq data, use ENA accession PRJEB47795:<br>Go to https://www.ebi.ac.uk/ena/browser/view/PRJEB47795<br><br>To review the novel ChIP-seq and AQuA-HiChIP data, use GEO accession GSE208146:<br>Go to https://www.ncbi.nlm.nih.gov/geo/query/acc.cgi?acc=GSE208146<br>Enter the token kpwngqykpxuzfel.
Files in database submission	# ChIP-seq in GEO<br>RH4_dCBP1_6h_H3K27ac_072523_CWRU_R1.fastq.gz<br>RH4_dCBP1_6h_H3K27ac_072523_CWRU_R2.fastq.gz<br>RH4_DMSO_6h_H3K27ac_072523_CWRU_R1.fastq.gz<br>RH4_DMSO_6h_H3K27ac_072523_CWRU_R2.fastq.gz<br>RH4-ABE_NT_Pol2_072523_CWRU_R1.fastq.gz<br>RH4-ABE_NT_Pol2_072523_CWRU_R2.fastq.gz<br>RH4-ABE_P3FC793R_Pol2_072523_CWRU_R1.fastq.gz<br>RH4-ABE_P3FC793R_Pol2_072523_CWRU_R2.fastq.gz<br>RH4_DMSO_6h_H3K27ac_072523_CWRU.nobl.bed<br>RH4_dCBP1_6h_H3K27ac_072523_CWRU.nobl.bed<br>RH4-ABE_NT_Pol2_072523_CWRU.nobl.bed<br>RH4-ABE_P3FC793R_Pol2_072523_CWRU.nobl.bed<br><br># HiChIP in GEO<br>RH4_P3F_FKBP12_DMSO_Pol2_HiChIP_071023_CWRU_R1.fastq.gz<br>RH4_P3F_FKBP12_DMSO_Pol2_HiChIP_071023_CWRU_R2.fastq.gz<br>RH4_P3F_FKBP12_dTag47_Pol2_HiChIP_071023_CWRU_R1.fastq.gz<br>RH4_P3F_FKBP12_dTag47_Pol2_HiChIP_071023_CWRU_R2.fastq.gz<br>RH4_DMSO_6h_Pol2_HiChIP_081721_NovoG_R1.fastq.gz<br>RH4_DMSO_6h_Pol2_HiChIP_081721_NovoG_R2.fastq.gz<br>RH4_A485_6h_Pol2_HiChIP_081721_NovoG_R1.fastq.gz<br>RH4_A485_6h_Pol2_HiChIP_081721_NovoG_R2.fastq.gz<br>RH4_dCBP1_6h_Pol2_HiChIP_081721_NovoG_R1.fastq.gz<br>RH4_dCBP1_6h_Pol2_HiChIP_081721_NovoG_R2.fastq.gz<br>RH4_P3F_FKBP12_DMSO_Pol2.allValidPairs.hic<br>RH4_P3F_FKBP12_dTag47_Pol2.allValidPairs.hic<br>RH4_DMSO_6h_Pol2_HiChIP_081721_NovoG.allValidPairs.hic<br>RH4_A485_6h_Pol2_HiChIP_081721_NovoG.allValidPairs.hic<br>RH4_dCBP1_6h_Pol2_HiChIP_081721_NovoG.allValidPairs.hic<br><br># RNA-seq in ENA<br>RH4_NT_072221_CWRU_R1.fastq.gz<br>RH4_NT_072221_CWRU_R2.fastq.gz<br>RH4_DMSO_072221_CWRU_R1.fastq.gz<br>RH4_DMSO_072221_CWRU_R2.fastq.gz<br>RH4_A485_072221_CWRU_R1.fastq.gz<br>RH4_A485_072221_CWRU_R2.fastq.gz<br>RH4_dCBP1_072221_CWRU_R1.fastq.gz<br>RH4_dCBP1_072221_CWRU_R2.fastq.gz<br>RH5_NT_072221_CWRU_R1.fastq.gz<br>RH5_NT_072221_CWRU_R2.fastq.gz<br>RH5_DMSO_072221_CWRU_R1.fastq.gz<br>RH5_DMSO_072221_CWRU_R2.fastq.gz<br>RH5_A485_072221_CWRU_R1.fastq.gz<br>RH5_A485_072221_CWRU_R2.fastq.gz<br>RH5_dCBP1_072221_CWRU_R1.fastq.gz<br>RH5_dCBP1_072221_CWRU_R2.fastq.gz<br>RH4_NT_072221_CWRU.gene.TPM.txt<br>RH4_DMSO_072221_CWRU.gene.TPM.txt<br>RH4_A485_072221_CWRU.gene.TPM.txt<br>RH4_dCBP1_072221_CWRU.gene.TPM.txt<br>RH5_NT_072221_CWRU.gene.TPM.txt<br>RH5_DMSO_072221_CWRU.gene.TPM.txt<br>RH5_A485_072221_CWRU.gene.TPM.txt<br>RH5_dCBP1_072221_CWRU.gene.TPM.txt

Genome browser session
(e.g. UCSC)

We viewed all datasets in IGV, not a public browser website. TDF files, a compressed version of BEDGRAPHS for IGV viewing, can be made available to reviewers if needed.

Methodology

Replicates

For AQuA-HiChIP experiments, the immunoprecipitation was performed in technical triplicate and pooled before library preparation, and biotin capture and library preparation were performed twice as independent duplicates and achieved near identical ratios of human to mouse contacts across all samples.

Sequencing depth

All ChIP-seq experiments were performed in pair-end read mode, 101 base pairs and were sequences to a depth of 40,000,000 Paired End reads. Spike in reads were measured in parallel mapping to dm3.
HiChIP-seq experiments were performed in pair-end read modes, 150 base pairs, and were sequenced to a depth of 350,000,000 Paired End reads. Spike in reads were measured in parallel mapping to mm10.
RNA-seq experiments were performed in pair-end read modes, 75 base pairs, and sequenced to a depth of 20,000,000 Paired End reads each.

#HiChIP_Sample_Name human_valid_contacts mm10_valid_contacts
RH4_A485_6h_Pol2_HiChIP_081721_NovoG 146995860 9932424
RH4_dCBP1_6h_Pol2_HiChIP_081721_NovoG 76785152 5024079
RH4_DMSO_6h_Pol2_HiChIP_081721_NovoG 102298703 7669289
RH4_P3F_FKBP12_DMSO_Pol2_HiChIP_071023_CWRU 43640994 10786562
RH4_P3F_FKBP12_dTag47_Pol2_HiChIP_071023_CWRU 55080565 10084237

#ChIP_seq_sample_name human_hg38_reads spikein_dm3_reads
RH4-ABE_NT_Pol2_072523_CWRU 67353436 6463875
RH4-ABE_P3FC793R_Pol2_072523_CWRU 61803294 7053873
RH4_dCBP1_6h_H3K27ac_072523_CWRU 65248627 4615532
RH4_DMSO_6h_H3K27ac_072523_CWRU 54164470 2463243

Antibodies

Novel experiments used anti-RNA Pol2 (Santa Cruz, cat. #sc-47701 X) and anti-H3K27Ac (Millipore, cat #MABE647 RM172).

Peak calling parameters

Peaks were called using MACS3 (version 3.0.0a6, https://github.com/macs3-project/MACS) using "default" mode for all targets reported in this paper, as they form sharp genomic peaks. Parameters for MACS3 usage: [--format BAMPE -B --pvalue 0.0000001]. Regions called as peaks which are known to be spurious mapping artifacts were removed before any further analysis (reference locations for sites black-listed by the ENCODE consortium, https://sites.google.com/site/anshulkundaje/projects/blacklists).

Data quality

Samples are listed with the number of peaks at a threshold of p < 1x10^(-7) in the 2nd column, and, in the 3rd column, the number of peaks with at least 5-fold or more enrichment are reported.
ChIP_seq_SampleFile PEAKS_PVALUE7 PEAKS_FOLD_CHANGE5
RH4-ABE_NT_Pol2_072523_CWRU 8256 7109
RH4-ABE_P3FC793R_Pol2_072523_CWRU 11371 10009
RH4_dCBP1_6h_H3K27ac_072523_CWRU 21529 7843
RH4_DMSO_6h_H3K27ac_072523_CWRU 34401 12206

Software

Fastq files were aligned to the human genome version hg38 using BWA version 0.7.17 and were visualized in IGV. The peak densities were calculated by igvtools after fragment and shifting correction and were normalized with dm3 (ChIP) or mm10 (HiChIP) spike-in following the previous study (PMID: 31784732). The significance of peaks was tested using MACS3 version 3.0.0a6.

Flow Cytometry

Plots

Confirm that:

☒ The axis labels state the marker and fluorochrome used (e.g. CD4-FITC).

☒ The axis scales are clearly visible. Include numbers along axes only for bottom left plot of group (a 'group' is an analysis of identical markers).

☒ All plots are contour plots with outliers or pseudocolor plots.

☒ A numerical value for number of cells or percentage (with statistics) is provided.

Methodology

Sample preparation

Cells used for flow cytometry were transduced with plamids for expression of either expressed blue fluorescent protein (BFP) or red fluorescent protein (RFP657). Two days after transduction, cells were mixed in a 1:1 ratio to follow their relative proliferation/survival rate. Before flow cytometric analysis, cells were detached from dishes, fixed with 0.5 % PFA for 5 min and washed with PBS.

Instrument

BD LSRFortessa Cell Analyzer

Software	For data collection, the BD FACSDiva 8.0.1 software was used. For data analysis the software FlowJo_V10.8.1 was used.
Cell population abundance	Both RFP657- and BFP-positive cells were 70-100% positive after transduction and therefore accounted for about 35-50% after mixing them in a 1:1 ratio.
Gating strategy	First, viable cells were gated based on FSC area /SSC area signal. Then singlet cells were gated based on FCS area/FCS height signals. Transduced cells expressing blue fluorescent protein or red fluorescent protein (RFP657) were selected based on fluorescence channels with the help of an untransduced control.

☒ Tick this box to confirm that a figure exemplifying the gating strategy is provided in the Supplementary Information.
